# Vitamin A-related nutrition knowledge gaps and predictors among caregivers of preschool children in Eastern Uganda: a cross-sectional study

**DOI:** 10.1186/s40795-024-00891-5

**Published:** 2024-06-11

**Authors:** Gilbert Mangusho, Eunice Njogu, Rhona Kezabu Baingana, Dorcus Mbithe David-Kigaru

**Affiliations:** 1 Department of Nutritional Science & Dietetics, Kyambogo University, P.O Box 1, Kyambogo, Kampala Uganda; 2 Department of Food, Nutrition & Dietetics, Kenyatta University, P.O Box 43844-00100, Nairobi, Kenya; 3 Department of Biochemistry & Sports Science, College of Natural Sciences, Makerere University, P.O Box 7062, Kampala, Uganda

**Keywords:** Vitamin A, Caregivers, Knowledge, Vitamin A deficiency, Sources, Signs/symptoms, Prevention, Preschool children

## Abstract

**Background:**

Vitamin A (VA) remains a core micronutrient as VA Deficiency (VAD) in children has persisted as a public health problem in parts of Africa with adverse effects. Caregivers of children are essential in the control of VAD; however, there is a paucity of data on their knowledge of VA, dietary sources, and VAD. This study sought to assess the level of VA-related nutrition knowledge (VANK) and its predictors among caregivers of preschool children in Eastern Uganda.

**Methods:**

A cross-sectional analytical design was used. Both socio-demographic and knowledge and attitude (KA) data were collected using a structured questionnaire partly adapted from the FAO model Knowledge, Attitude and Practice (KAP) questionnaire. A sample size of 256 was used. Caregivers of 24–59 months-old children were selected from Bukwo District in Eastern Uganda using purposive and random sampling methods. Knowledge scores (%) based on responses to ten questions were determined and eventually classified as low (≤ 40%) and moderate or high (˃40%). Descriptive and inferential statistics were computed using SPSS (version 24). Logistic regression was used to identify predictors with *p* < 0.05 considered significant.

**Results:**

The study had 247 caregivers with a mean age of 30.9 ± 7.7 years. The majority were female (90%), married, subsistence crop farmers and had primary-level education or lower. The mean VANK score was 18.9 ± 24.7%. Overall, most of the caregivers had low VANK as only about 20% had moderate or high. The proportions that knew the different aspects of VANK were correspondingly small. About half of the caregivers (46.6%) knew VA itself and only 27% knew any of its sources. Those who knew VAD, its causes, signs/symptoms and prevention measures were 31, 22, 13 and 24% respectively. The caregivers’ VANK was significantly associated with their overall VA-related attitude, age and level of education. However, education and age were the significant predictors.

**Conclusion:**

Caregivers had very low VANK. They barely knew VA and its food sources or VAD. The main predictors of VANK were caregiver age and level of education. The study recommends education of caregivers about VA for effective VAD control which contributes to achievement of the Sustainable Development Goal (SDG) 2.

**Supplementary Information:**

The online version contains supplementary material available at 10.1186/s40795-024-00891-5.

## Background

Vitamin A Deficiency (VAD) is linked to poverty, especially in Sub-Saharan Africa (SSA) [1, 2] and is persistent in Uganda and other low-income countries (LICs) in Africa, South Asia, and the Caribbean [3, 4]. In these countries, sub-clinical VAD affects 6–35% of preschool children [5, 6]. Worldwide, it is estimated that over 200 million preschool-age children have VAD [7] and it remains a public health problem, particularly in SSA [8]. The primary effects of VAD are the functional impairment of sight and debilitation of various physiological processes including the immune response and wide-ranging secondary effects on health, productivity and growth of populations [1, 6].

Despite nearly three decades-long VAD control efforts in Uganda including vitamin A supplementation (VAS), food fortification, and bio-fortification [9–11], VAD remains a significant problem. The leading cause of VAD in preschool-age children is poor dietary vitamin A (VA) intake [12]. This factor could be addressed if caregivers (persons primarily charged with daily preschool-child care/feeding) had the requisite knowledge, skills and material resources. Caregivers’ VA-related nutrition knowledge (VANK), including knowledge of VA as a nutrient important for the sight function and of VAD as a physiological disorder resulting from insufficient bodily VA, facilitates the development of useful attitudes and practices that aid in alleviating the VAD burden [1, 13].

## Study design and setting

This was a cross-sectional analytical study involving caregivers of preschool children aged 24–59 months. It was carried out in Bukwo District in Eastern Uganda, a region with a total population of 9,042,422 [14], one of the poorest [15] in addition to having higher rates of VAD than most of the other regions in Uganda. The main economic activity is subsistence agriculture, similar to other rural areas in the country [16]. Bukwo Districts is located on the eastern slopes of Mt Elgon, with the administrative centre about 130 and 350 km northeast of Mbale and Kampala respectively. It borders the districts Kween (to the west and northwest) and Amudat (north), and the Republic of Kenya to the east and south. The district, unlike most other parts of the country has one main crop-growing season in a year where, typical of Uganda, a variety of crops mainly maize and beans are grown. At the time of the study, the district was minimally accessible due to poor roads and mountainous terrain. The nearest all-weather road was about 70 km away.

### Sample selection

The sample size (n) was first calculated from the Yamane formula n = N/(1 + N(e)^2^) [17], where N was the estimated population of preschool-age children in Eastern Uganda (1.3 million [14]) and e 5%. The result (400) was then modified for cluster sampling according to Hemming et al. [18] and Killip et al. [19]. An intra-class correlation coefficient (ρ) of 0.03 was assumed and the required cluster number (nρ or 12) was raised to 32 (by 20) for higher power and reasonable cost. Therefore, the maximum cluster size would be 20 (n/20) and corresponding sample size 640. However, preliminary qualitative data showed that a typical cluster in the area had only 6–10 eligible caregivers. Considering the available resources, a cluster size of eight, and 32 clusters were finally chosen, hence a total sample size of 256.

**Fig. 1:**
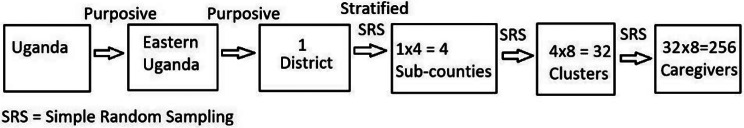
Sampling procedure

A Multi-stage procedure (Fig. 1) was used to select the caregivers. Both Eastern Uganda and Bukwo District were purposively selected, the former for its relative share of VAD, and the latter for being largely rural, remote from large food markets and lacking evidence of on-going VA-related interventions. To select caregivers, the district (12 sub-counties) was geographically stratified into four equal blocks from each of which one sub-county was selected using Simple Random Sampling (SRS)/lottery. A list of clusters/villages with estimated sizes was obtained from the respective sub-county leaders. Small clusters (< 25 households) were excluded because they were more unlikely to provide the required numbers of caregivers. Eight clusters and, subsequently, eight households/caregivers with children 24–59 months were selected by SRS (lottery). The main individual that was responsible for making the daily child-feeding decisions in a household was chosen as the caregiver. Those who had spent less than a month with the child, had mental illnesses, or did not consent to the study were excluded.

### Data collection

Data were collected using a structured questionnaire. The data consisted of VA knowledge (including VA itself, food sources, and VAD, its causes, signs/symptoms and prevention), VA-related attitude and socio-demographics (gender, age, education, marital status, household size, occupation, economic activity, income, caregiver-child-household head relationships, type of health-care provider). The questionnaire (see Supplementary File 1) was partly adapted from FAO’s Knowledge, Attitude, and Practices (KAP) model questionnaire and guidelines [16]. The model questionnaire was chosen because of its uniqueness in having a module specific for VA which was appropriate for this study. The final version contained 10 mostly open-ended knowledge questions, eight attitude items based on the Health Belief Model (HBM) captured using a five-point Likert scale as shown in Table 1, and a number of socio-demographic questions adapted from USAID’s Demographic and Health Survey (DHS) program model questionnaires.

**Table 1:** Likert scale values for measurement of VA-related attitude variables

Attitude Variable*	Value on Likert scale**				
	1	2	3	4	5
Susceptibility to VAD	Very unlikely	Unlikely	Neutral	Likely	Very likely
Severity/seriousness of VAD	Not serious at all	Not serious	Neutral	Serious	Very serious
Importance of feeding child with VA foods	Not important at all	Not Important	Neutral	Important	Very important
Taste of VA-rich plant foods	Dislike greatly	Dislike	Neutral	Like	Like greatly
Difficulty preparing VA foods	Very difficult	Difficult	Neutral	Not difficult	Not difficult at all
Confidence preparing VA foods	Not confident at all	Not confident	Neutral	Confident	Very confident
Cues for feeding VA foods	Disagree strongly	Disagree	Neutral	Agree	Agree strongly
Barriers to feeding VA foods	Strongly Disagree	Disagree	Neutral	Agree	Strongly Agree

*As perceived by caregiver; **Responses ranked on a scale of 1–5

The enumerators were recruited and trained before the study. The questionnaire was translated into the local language and then back-translated to English during training, pretested and adjusted accordingly before being used for the actual data collection. Face-face interviews with caregivers were conducted at the household premises using the local language.

### Data analysis

Data were manually checked daily during collection to ensure completeness. Responses to knowledge questions were later scored using a marking guide designed to control for guesswork. Answers to questions especially the multiple-response type such as VA sources, and causes, signs/symptoms and measures to prevent VAD were scrutinized based on their consistency. Each correct answer was awarded 2 marks and an incorrect one, 0 (zero). The data were then entered using an MS Access 2016 (Version 16.0) database equipped with validation rules to limit errors during entry. The entered data were inspected, cleaned, and exported to SPSS (Version 24) for further cleaning and analysis.

The scores for all knowledge items were summed up to obtain an individual caregiver mark which and converted into a percentage. The maximum possible score (100%) was 20 marks. The mean score (%) was computed for all caregivers. The scores were also categorized into low (≤ 40%), moderate (41–69%) and high (≥ 70) as in Kigaru et al. [20]. The lower cut-off of 40% was considered appropriate to distinguish no or low from reasonable knowledge for ordinary caregivers who had no prior preparation for this relatively more specific study (on VA) as there were no similar studies or references. For attitude, an aggregate score was obtained for each caregiver by calculating the mean score in the eight items. The mean aggregate score was also computed to represent the overall caregivers’ attitude. Aggregate scores ≤ 3 were categorized as poor attitude whereas 4 and 5 were categorized as good. Proportions of caregivers with good and poor attitude were established. The SPSS (Version 24) and MS Excel 2016 (Version 16.0) were used to perform statistical tests and data transformations. Descriptive statistics including frequencies, means (and standard deviations) were computed and used to describe the data. Bivariate and multivariate binary logistic regression analyses were performed to identify the predictors of VANK. In the regression, the VANK categories were reduced to two by creating a dichotomy around the lower cut-off, in effect, merging “moderate” and “high.” Because attitude data were obtained from only a sub-sample, attitude was excluded from the multivariate analysis. The unit of analysis was the individual caregiver rather than the cluster to maintain the study power.

## Ethical approval

This study was approved by the Mildmay Uganda Research Ethical Committee, MUREC (REC REF 0306–2019) and registered by the Uganda National Council for Science and Technology, UNCST (HS2664). Before inclusion, the caregiver provided written consent after an explanation of the study aims and procedures had been made to them. The filled questionnaires were kept in a special locked room and coded before entry to remove any personally identifiable information to ensure anonymity and confidentiality.

## Results

### Socio-demographic characteristics of caregivers

A total of 247 out of the 256 selected caregivers participated in the study, representing nearly 97% of the sample size. Only 3% were not found. The overall mean age was 30.9 ± 7.7 years, and most of them (90%) were female. The majority had, at most, primary school-level education, were married (94.3%), biological parents to the children (93%), subsistence crop farmers, and earned low monthly household income ($14–28) as shown in Table 2. The mean household size was 6.4 ± 2.2 persons (95% CI: 6.2–6.7).

**Table 2:** Caregiver and household characteristics

**Caregiver/Household Characteristic**	*n*	%
**Age (years)**		
≤ 25	71	28.7
26–30	70	28.3
31–35	40	16.2
≥ 36	66	26.7
Education*		
Primary and below	143	57.9
Lower secondary	86	34.8
Upper secondary and beyond	18	7.3
**Occupation**		
Household chores	83	33.6
Subsistence farmer	98	39.7
Small business owner	48	19.4
Civil servant	18	7.3
**Household size**		
Small (≤ 4 persons)	92	37.3
Medium (5–7 persons)	137	55.4
Large (≥ 8 persons)	18	7.3
Household monthly Income (UGX**)		
< 50,000	52	21
50,000-<100,000	80	32.4
100,000-<250,000	56	22.7
≥ 250,000	59	23.9
**Home/kitchen garden operated?**		
No	17	6.9
Yes	230	93.1
**Household’s Health-care provider**		
Government hospital	69	27.9
Government Health Centre	168	68
Private clinic	10	4

*Highest level of education attained by a caregiver; **1 UGX ≈ $1/3,600; n = number/count; %=percent

### Overall vitamin A-related knowledge

The caregivers had very low levels of VANK (mean = 18.9%±24.7, 95% CI: 15.9–21.9). More than half (132/53.4%) had no VANK whatsoever (VANK = 0). Among those who had some knowledge (VANK > 0), the mean VANK score (40.6 ± 20.7%, 95% CI: 37.0-44.3) was marginally moderate and, therefore, shallow. A clear majority of all the caregivers (79.4%, 95% CI: 74.5–84.2) had low VANK. The rest had moderate (15.8%, 95% CI: 11.3–20.6) or high (4.8%, 95% CI: 2.4–7.3). Therefore, only about 20% had moderate or high knowledge and the rest, low. There was no difference in VANK between the clusters, x^2^(31) = 42.64, *p* = 0.08.

### Knowledge of vitamin A, sources, and deficiency

**Fig. 2:**
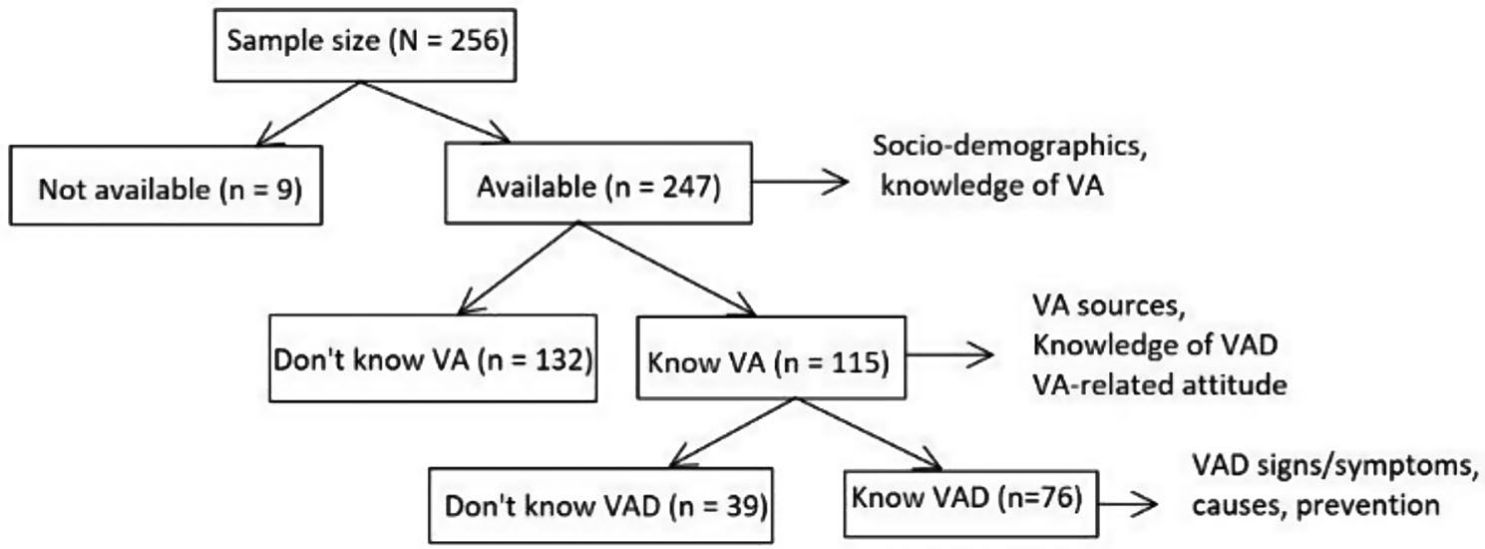
Flow diagram for sub-sample sizes for different aspects of VANK and VA-related attitude

Barely half of the caregivers (46.6%) knew VA itself and were therefore able to provide data on VAD and VA-related attitude (Fig. 2).

Small proportions of caregivers had knowledge of the different aspects of VA (Table 3). Less than a third of all (two-thirds of those who knew VA) knew VAD. Among them, the majority correctly identified the causes and preventive measures for VAD. There were also wide gaps in knowledge of VA-rich foods in the different groups (animal; Dark Green Leafy Vegetables, DGLVs; fruits; Vegetables, Tubers and Roots, VTRs, and VA-fortified foods) as shown. Animal-food sources were the best-known while VA-rich VTRs were the least. The VA-fortified foods were much better known than the DGLVs and VA-rich fruits.

**Table 3:** Caregiver knowledge of different aspects of VA and VAD

Aspect of VANK	Caregivers with knowledge		
	*n*	% (*N* = 115 or 76)^1^	% (*N* = 247)^2^
**VA ** **Sources and VAD**		N = 115*	
Animal foods	67	58.3	27.1
DGLVs	19	16.5	7.7
Fruits	7	6.1	2.8
VTRs	3	2.6	1.2
VA-fortified foods	36	31.3	14.6
VAD	76	66.1	30.8
**VAD** ** signs, causes, prevention**		N = 76**	
Signs/symptoms of VAD	32	42.2	13.0
Causes of VAD	54	71.1	21.9
Prevention of VAD	60	78.9	24.3

VA = Vitamin A; VAD = Vitamin A Deficiency; ^1^Caregivers who knew VA or VAD; ^2^All caregivers

*Caregivers who knew VA; **Caregivers who knew VAD; DGLVs = Dark Green Leafy Vegetables; VTRs = Vegetables, Tubers and Roots

As shown in Table 4, the caregivers mentioned various foods within the different groups as sources of VA. The leading foods were eggs and dairy (animal), Dodo (*Amaranthus*), passion fruits, Irish potatoes (plant) and cooking oil and wheat flour (industrially processed). However, some of the foods, such as passion fruits and potatoes were not truly good VA sources. The best known true VA-rich foods among the fruits and VTRs were actually ripe mango and orange-fleshed sweet potato respectively, both mentioned with incomparably lower frequencies than the non-VA rich counterparts.

**Table 4:** Knowledge of VA-rich foods

VA-rich Food*	Caregivers		
	**n**	**%(N = 115** ^**a**^ **)**	**%(N = 247** ^**b**^ **)**
**Animal foods**			
Liver	39	33.9	15.8
Eggs	62	53.9	25.1
Milk/Milk products	62	53.9	25.1
Others (non-VA-rich)^d^	26	22.5	10.5
**DGLVs**			
Kale *(Brassica spp)*	51	44.3	20.6
Amaranthus leaves/dodo	52	45.2	21.1
Pumpkin leaves	17	14.8	6.9
Local green vegetables (various)	43	37.4	17.4
Others (non-VA-rich vegetables e.g., cabbages)^c^	26	22.6	10.5
**Fruits**			
Ripe mango	18	15.7	7.3
Ripe papaya	5	4.3	2.0
Others (non-VA-rich: melons, oranges, berries, guavas, passion fruits, etc.) ^c^	120	104.3	48.6
**VTRs**			
Orange sweet potato	17	14.8	6.9
Carrot	11	9.6	4.5
Pumpkin	5	4.3	2.0
Others (non-VA-rich potatoes, yams, etc.)^c^	93	81	37.7
**VA-fortified**			
Margarine	15	13.0	6.1
Cooking oil	23	20.0	9.3
Wheat Flour	23	20.0	9.3
Others (not VA-fortified)^c^	84	73.1	34.0

^a^Caregivers who knew VA; ^b^All caregivers; ^c^Foods mentioned but incorrect: DGLVs = Dark Green Leafy Vegetables; VTRs = Vegetables, Tubers and Roots; *according to caregivers; n = number/count; % = percent

### Knowledge of causes, signs/symptoms, and prevention measures for VAD

The majority of caregivers mentioned consumption of a poor variety of food as the cause of VAD (Table 5). Other causes cited included a VA-deficient diet, infections, inadequate food intake, poor feeding, poor fruit intake, inadequate breastfeeding, and insufficient medical care. Among the VAD signs/symptoms mentioned, physical weakness (an unspecific symptom) predominated over eye problems and frequent infections which are the key VAD signs/symptoms. Also, the leading VAD control measure named by caregivers was increased consumption of VA–rich (including VA-fortified) foods.

**Table 5:** Causes, signs/symptoms, and means of prevention of VAD by caregivers

Aspect of VAD		Caregivers			
		**n**	**%(N = 76)** ^**1**^	**%(N = 247)** ^**2**^	
**Causes**					
Poor food variety		47	61.8	19	
Diet lacking VA		5	6.6	2	
Infections		5	6.6	2	
Too little food		8	10.5	3	
Others (Poor feeding, fruit intake, breastfeeding and medical care)		8.9	11.7	3.4	
Don’t know		9	11.8	4	
**Signs/Symptoms**					
Physical weakness		48	63.2	19.4	
Eye problems		10	13.2	4.1	
Infections		22	29	8.9	
Don’t know		16	21	6.5	
**Preventive measures**					
VA-rich diet		50	65.7	20.2	
VA-supplements		14	18.4	5.7	
Nutritious and varied diet		8	10.4	3.2	

^1^Caregivers who knew VAD; ^2^All caregivers; n = number/count; % = percent

### Vitamin A-related attitude

The mean aggregate attitude score among caregivers who knew VA itself was 4.03 ± 0.52 (95% CI: 3.93–4.13) and the majority had a good attitude (90.4%).

### Predictors of vitamin A-related knowledge

The independent variables (age, sex, marital status, education level, occupation, overall attitude, household size, income, home garden operation, and type of health-care provider) were included in binary logistic regression analysis models to establish their effect on VANK. Crude and adjusted odds ratios (COR and AOR) were used to represent the relationships. Caregiver’s age, education and VA-related attitude had statistically significant CORs (Table 6). Only age and education were included in the final model because data were available for the entire sample unlike for attitude which were drawn from a subset. Inclusion of attitude would therefore adversely affect the model. The AORs for both age and education were statistically significant (*p* < 0.05).

**Table 6:** Logistic regression for predictors of VANK

Variables	Caregiver VANK (%)		COR(95% CI)*p*-value		AOR(95% CI)*p*-value
	**Low**	**Mo-Hi**			
**Sex of caregiver**					
Male	18(7.3%)	7(2.8%)	1		
Female	177(71.7%)	45(18.2%)	0.654(0.257–0.661)0.372		
**Age of Caregiver (years)**					
≤ 25	58(23.5)	13(5.3)	0.516(0.232–0.145)0.104		0.528 (0.231–1.207)0.130
26–30	60(24.3)	10 (4.0)	0.383 (0.164–0.898)0.027*		0.384 (0.160–0.920)0.032*
31–35	31 (12.6)	9 (3.6)	0.668 (0.269–1.657)0.384		0.639 (0.251–1.630)0.349
≥ 36	46(18.6)	20 (8.1)	1		1
**Caregiver’s Education**					
≤Primary	118(47.8%)	25(10.1%)	1		0.219 (0.078–0.620)0.004*
Lower secondary	68(27.5%)	18(7.3%)	1.249(0.636–0.455)0.518		0.308 (0.104–0.912)0.033*
≥Upper secondary	9(3.6%)	9(3.6%)	4.720(1.702–3.086)0.003*		1
**Marital status**					
Married	182(73.7%)	51(20.6%)	3.643(0.465–28.511)0.218		
Single	13(5.3%)	1(0.4%)	1		
**Household size**					
Small^1^	73(29.6%)	19(7.7%)	1		
Medium^2^	110(44.5%)	27(10.9%)	0.943 (0.489–1.820)0.861		
Large^3^	12(4.9%)	6(2.4%)	1.921(0.638–5.785)0.246		
**Caregiver’s occupation**					
Household chores	65(26.3%)	18(7.3%)	1		
Subsistence farmer	76(30.8%)	22(8.9%)	1.045(0.516-2.117)0.902		
Small business	41(16.6%)	7(2.8%)	0.617(0.237-1.605)0.322		
Civil servant	13(5.3%)	5(2.0%)	1.389(0.437-4.413)0.578		
**Monthly household Income (UGX)**					
< 50,000	43(17.4%)	9(3.6%)	1		
50,000 -<100,000	67(27.1%)	13(5.3%)	0.927(0.365–2.355)0.873		
100,000 -<250,000	42(17.0%)	14(5.7%)	1.593(0.623–4.074)0.331		
>=250,000	43(17.4%)	16(6.5%)	1.778(0.709–4.459)0.220		
**Attitude**					
Poor	10(8.7%)	1(9.6%)	1		
Good	53(46.1%)	51(44.3%)	9.623(1.189–77.897)0.034*		
**Home garden operated?**					
No	16(6.5%)	1(0.4%)	1		
Yes	179(72.5%)	51(20.6%)	4.559(0.590-35.203)0.146		
**Health care provider**					
Government hospital	59(23.9%)	10(4.0%)	1		
Government Health center	128(51.8%)	40(16.2%)	1.844(0.864–3.936)0.114		
Private clinic	8(3.2%)	2(0.8%)	1.475(0.273–7.980)0.652		

COR = Crude Odds Ratio; AOR = Adjusted Odds Ratio; ^1^≤4 persons; ^2^5-7 persons; ^3^≥8 persons; Mo-Hi = Moderate or high; *p-value is significant

As shown, caregivers with higher levels of education (≥ upper secondary) were more likely to have moderate or high VANK compared to those with lower levels or none (*p* < 0.05). Similarly, older caregivers (≥ 36 years) were more likely to have moderate or high knowledge than younger ones aged 26–30.

## Discussion

The purpose of this study was to establish the gaps in and predictors of VANK among caregivers of preschool children in Eastern Uganda. The level of VANK among caregivers was found to be low (only nearly 20%), with a clear majority (about 80%) having low knowledge. More than half of the caregivers did not know VA itself; around 75% had no knowledge of any VA-rich food or VAD and its signs/symptoms, causes and control measures. Two factors: caregiver’s age and educational level were significant predictors of VANK.

The study was carried out in Bukwo district which, despite being relatively inaccessible, has largely similar socio-demographic/economic and agro-ecological characteristics to those of most rural areas of other districts in Eastern Uganda [14, 21]. The caregivers were mainly youthful married mothers of children 24–59 months, representing a mature and experienced cohort that is active in child feeding. They were slightly older than those for a similar study in Pakistan (29 years) [22], and younger than for another one in Tanzania (40.75 years) [23]. The households, like most in Eastern Uganda, were larger than the average nationally (4.6 persons) and in rural Uganda (4.8 persons) [16]. Compared to national statistics on educational attainment for aggregate national, rural or mountainous regions [16] (the study site being in a mountainous area), the caregivers had levels of education which were similarly distributed, as the highest proportion was those with primary education or none followed by those with some secondary education and the lowest, those with upper secondary and beyond.

The main instruments adapted for this study have been used by others including Weerasekera et al. [24]. They are based on other validated tools/models such as the HBM [25] and are generally considered valid. In addition, the module for VA was peculiarly relevant to this study as similar modules were rare or unavailable.

This study found that most caregivers had low VANK. A systematic review by Barbosa et al. [26] shows that knowledge is commonly classified into low, moderate, and high although some studies, such as Liu et al. [27], do not explicitly do so. Most studies, including Kigaru et al. [20], which used the knowledge classification were, however, concerned with general nutrition knowledge and not specifically on VA as this study was, and involved different population groups. Similar studies were rare and, therefore, a reference cut-off was not available. The 40% cut–off used in this study facilitated a more realistic assessment of the specific kind of nutrition knowledge among a group with widely varying characteristics.

These findings were similar to those in other related studies [22, 28] but slightly different from studies in Tanzania [23], Kenya [29], Ghana [30] and Ethiopia [31] which showed higher knowledge levels. These studies were different from this study because most of them involved urban caregivers who are usually more informed than rural dwellers, were conducted after major interventions, and concerned mainly VAS.

The caregivers had generally poor knowledge of most of the aspects of VANK, but there were some variations within different aspects. On VA sources, animal-foods were known better than other food types possibly due to a seemingly narrow range of animal foods used by the caregivers which incidentally are also good VA sources. Some of these foods including the eggs and dairy, are cheaper and therefore more frequently used than others, for example, liver. The fortified foods are equally few and usually labelled and promoted on mass media channels hence increasing caregivers’ knowledge which, however, was still very low. Hardly 10% of all caregivers knew any VA-rich fruit, vegetable or root/tuber, showing that they lacked basic knowledge of the nature of VA-rich plant foods in which the orange colour as seen in ripe mangoes, pumpkins and carrots, is a key indicator [32]. The DGLVs like kale and Amaranthus, though excellent pro-VA sources [28, 33], were not clearly known. Despite being mentioned, they were often undistinguished from cabbages, beans and other non-VA-rich vegetables thereby portraying a lack of true knowledge. Similarly, VA-rich fruits, roots and tubers were less known. In addition, it appeared that majority of caregivers incorrectly believed that any fruit was a good source of VA.

There was little knowledge of VAD among caregivers. This knowledge was considered in this study to be contingent on that of VA itself although it may not always be true. Indeed, a large proportion of caregivers who knew VA did not know VAD. This study, together with others [22, 28], reveals serious gaps in knowledge of VAD across different societies in LICs and calls for stronger educational action. Consumption of a poor variety of food topped the caregivers’ causes of VAD while inadequate dietary VA intake, a more direct and presumably a readily discernible cause, was of negligible proportion.

Eye problems (physical and functional defects) are the classic VAD signs/symptoms [28, 34] unlike frequent infections and variable skin conditions [35] which are non-specific. Infections have a vicious relationship with VA [35, 36]. The ability of caregivers to correctly recognize VAD in children is essential in its control, however, they were scarcely competent. These findings are consistent with those of Hadzi et al. [30] in exposing the danger of the hidden but prevalent sub-clinical VAD [5, 21].

Finally, caregiver participation in VAD prevention is recommended. Although fortunately some caregivers knew appropriate prevention measures, the limited knowledge of both VAD (Table 3) and the right foods (Table 4) would be a discount. Improving the knowledge of VA-rich foods and VAD among caregivers potentially narrows this gap. Use of VA supplements and a nutritious diet, among other methods mentioned, are effective but problematic due to the former’s high cost and non-food nature [37, 38] and poor understanding of the latter.

### Predictors of VANK

In this study, age of caregiver and educational attainment were found to be predictive of VANK. Level of formal education attained was a positive predictor of VANK. This was in agreement with other studies [13, 26, 27]. Education was indicated according to formal educational categories, which reflected to years of schooling: primary and below (0–7), lower secondary (8–11), upper secondary and beyond (≥ 12). This study strengthens the others which suggest that formal education directly or indirectly contributes to health-related knowledge through increased exposure and ability to comprehend and apply pertinent information. Generally, maternal education correlates positively with child survival through better healthcare practices [39], and knowledge could play an important intermediary role.

Concerning age, caregivers above 35 had greater odds of moderate or high VANK than those who were a decade younger. These findings show that a ten-year age difference among caregivers of preschool children is significant in terms of VANK and point to a role of experience in child care. The senior caregivers have possibly had 3–10 more rounds of pre-school child-care experience than the junior ones. It’s argued that accumulated experiences increase particular knowledge [40]. Accordingly, older caregivers are wont to possess greater knowledge than their juniors due to their longer participation in, for example, the national twice-yearly VAS programme, interaction with the healthcare system/professionals and peers, and self-discovery through the child-feeding chores.

### Strengths and limitations

This study concerns caregivers’ knowledge of VA, a key nutrient for the health and growth of children. Studies in this area are quite rare. Caregivers are essential in children’s nutrition, and their knowledge of VA is crucial. The findings from this study are important for the effective engagement of caregivers for the prevention of VAD among children with the potential of reducing the reliance on expensive VAS. However, there were some limitations. The scope did not fully cater for the wide ethnic and cultural diversity in Eastern Uganda although the sociodemographic and economic characteristics are largely the same for rural areas in Uganda. Also, only caregivers who knew or had heard of VA were asked about VAD, thereby excluding data from caregivers who probably knew VAD independently of VA. The study relied on caregivers for estimation of certain variables such as education level, age, and income and could not therefore control the level of precision even though deep probing was employed in data collection. Finally, the power of the study was limited by the small sample size. However, stratified sampling and individual-level analysis made the study stronger.

## Conclusion

The objectives of this study were to assess the VA-related nutrition knowledge (VANK) and identify its gaps and predictors among caregivers of preschool-age children. The caregivers possessed very low (only about 20%) VANK. Therefore, there was an 80% gap in VANK among caregivers of preschool children aged 24–59 months. More than half of caregivers had no VANK whatsoever. There was a lack of deep knowledge of VA as only nearly a quarter of all caregivers knew any other aspect concerning VA including VA/pro-VA-rich foods and causes, signs/symptoms and prevention of VAD. The key predictors were caregiver’s age and educational level and VANK appeared to increase with both. This study recommends deliberate efforts to promote knowledge of VA and related aspects among caregivers of preschoolers in rural areas where VAD is a problem. More attention is needed for the younger and less-educated caregivers to narrow the gaps in VANK. Enhanced VANK among caregivers will strengthen VAD control and contribute to the achievement of SDG 2 and other nutrition-related goals. More studies, including the formulation and regularization of precise nomenclature for VA and related aspects in local dialects, are needed to further investigate VANK predictors among caregivers in varying contexts and also to improve VAD control policy formulation and effectiveness.

### Electronic supplementary material

Below is the link to the electronic supplementary material.

Supplementary Material 1

Supplementary Material 2

Supplementary Material 3

## Data Availability

The data used for this manuscript are obtainable upon reasonable request.

## Abbreviations

FAO: Food & Agriculture Organization
HBM: Health Belief Model
KAP: Knowledge Attitudes and Practices
LC: Local Council
LIC: Low-Income Country
MUREC: Mildmay Uganda Research Ethical Committee
SDG: Sustainable Development Goal
UNCST: Uganda National Council for Science & Technology
VA: Vitamin A
VAD: Vitamin A Deficiency
VANK: Vitamin A-related Nutrition Knowledge
VAS: Vitamin A Supplementation
DGLVs: Dark Green Leafy Vegetables
VTRs: Vegetables, Tubers and Roots
