# Supplementary material for: Vitamin A-related nutrition knowledge gaps and predictors among caregivers of preschool children in Eastern Uganda: a cross-sectional study
Source: BMC Nutr. 2024 Jun 11;10:85. doi: 10.1186/s40795-024-00891-5 (PMC11167797; doi:10.1186/s40795-024-00891-5)
Supplement: Supplementary file 1 — Supplementary Material 1 [file 40795_2024_891_MOESM1_ESM.docx]

**Questionnaire for Caregivers**

**To be administered at the household**

**SECTION A: Household Socio-economic and demographic Information**

Questionnaire ID:____________ Interview date:________________ Interviewer:_________________

Sub-county:_________________ Parish: _______________ Village/cluster:______________

Household ID:_______________ Child ID:______________ Caregiver ID:________

1. **Caregiver and General Household characteristics**

**H01: Caregiver characteristics**

| **H01.1** Sex | **H01.2** Age (yrs) | **H01.3** Household head? | **H01.4** Relationship to Hhld head | **H01.5** Marital status | **H01.6** Have you ever attended school? | **H01.7** Highest level of school you have attended? | **H01.8** What is your primary Occupation? |
| --- | --- | --- | --- | --- | --- | --- | --- |
|  |  |  |  |  |  |  |  |
| 1= Male  2=Female |  | 1 = yes  0 =No | 1=spouse  2=son/daughter  2=Brother/sister  3=niece/nephew  4=self  5=Sister/brother in-law  6=other (specify) | 1=Married (monogamous)  2=Divorced/separated  3=Widowed  4=Single  5. polygamous married | 1 = yes  0 =No | 0=None  1=Primary  2=”O” level  3=”A” level  4=Tertiary  5=University  6=FAL | 1=Housewife/stay-home parent  2=subsistence crop farmer  3=Commercial crop farmer  4=casual labour  5=businessman/woman  6=civil servant  7=Private sector employee  8=volunteer (formal)  9=other(specify) |

***H02.* Household demographic information**

I am going to ask you about other members of your household. How many are:

|  | **Male (number)** | **Female (number)** | **Total (number)** |
| --- | --- | --- | --- |
| ***H02.1*** 0-5 months? |  |  |  |
| ***H02.2*** 6-23 months? |  |  |  |
| ***H02.3*** 24-59 months? |  |  |  |
| ***H02.4*** 6-12 years? |  |  |  |
| ***H02.5*** 13-17 years? |  |  |  |
| ***H02.6*** 18 years and above? |  |  |  |
| ***H02.7*** Total |  |  |  |

**H03-18. Household Nutrition and other socioeconomic characteristics**

***H03***. What is the main economic/livelihood activity in this household? ______

***1****=Crop farming* ***2****=Livestock farming* ***3****=Trade in produce* ***4****=Hospitality* ***5****=Petty trade* ***6****=Salaried employment; 7=Don’t know;* ***8=Others (specify)***

***H04***. What is the average total income for this household in one month (UGX)? _______

***1****=0-<50000;* ***2****=50,000-<100,000;* ***3****=100,000-<250,00;* ***4****=250,000-<500,00;* ***5****=500,000-1,000,000;* ***6****=>1,000,000;* ***7****=Don’t know*

***H05.*** What is the staple food (carbohydrate) in your household? _______

*1=Posho; 2=Matooke 3=Millet ; 4=Cassava; 5=Rice; 6=Sweet potato; 7=Other (specify)*

***H06***. What is the most common food (sauce) consumed in your household? ______

*1=Beans; 2=meat; 3=Fish; 4=chicken; 5=Kale (Sukuma wiki); 6=cabbage;7=Groundnuts; 8=Local green vegetables (specify); 9=Others (specify)*

***H07***. How many meals do you have in this household in a day? ______

***H08***. Where do you and your family go for health care? ______

*1=Government Hospital; 2=Government Health Center; 3=Private hospital; 4=Private clinic/medical center 5=Family Planning Clinic; 6=Mobile Clinic; 7=Other Public Sector; 8=Other (specify)*

**SECTION B: Vitamin A-related Knowledge**

I am going to ask you some questions about vitamin A (nutrient for sight) and food rich in vitamin A. Please let me know if you need me to clarify on any of my questions. Feel free to ask any question you may have.

**K.1.** Have you heard/Do you know about vitamin A (nutrient that enables proper sight)? ***(0=*** *No****, 1=****Yes* ) ***_______ If no, End.***  __ _____

*Preliminary analysis:*

**Marks** *( 0=No; 2=Yes)* **_____**

**K.2** Have you heard about vitamin A deficiency or lack of enough vitamin A in the body? **(*Probe!****) (1= yes; 0=No/don’t know)* ***__***____  ***If no, skip to K.6***

*Preliminary analysis:*

Marks *(0= No/don’t know; 2=yes)* _____

**K.3** If Yes in K.2: Can you tell me how you can recognize someone who lacks vitamin A in his or her body/has vitamin A deficiency? (List *all*) ***__***____ ***1****. Weakness/feels less energetic;* ***2****. be more likely to become sick /sickly (less immunity to infections);* ***3****. Eye problems: night blindness (inability to see at dusk and in dim light), dry eyes, corneal damage, blindness;* ***4****. Other;* ***5****. Don’t know*

*Preliminary analysis:*

Number of correct responses ______

Knows/Doesn`t know ______ (1= Does not know; 2= Knows - at least 1 correct response)

**Marks** *(0 = Does not know; 2= Knows)* **_____**

**K.4**. In your opinion, what causes a lack of vitamin A in the body? (list all) ______

***1.*** *Poor variety of foods;* ***2****. Eating too little food/not eatting much (poor intake);* ***3****. Eating foods poor in vitamin A content;* ***4****. Diseases e.g. malaria;* ***5****.Having no oil in the food;*  ***6****.Worm infestations;* ***7****.Other;* ***99****. Don’t know*

*Preliminary analysis:*

Number of correct responses ____

Knows/Doesn`t know ____*(1= Doesn’t know; 2= Knows - at least 1 correct response)*

**Marks** *(0 = Does not know; 2= Knows*) ***_____***

**K.5**: How can one prevent a lack of enough vitamin A in the body? _ _ _____

***1****. Eat/feed vitamin-A-rich foods – having/giving a diet rich in vitamin A:* ***2****. Eat/feed foods fortified with vitamin A;* ***3****. Give vitamin A supplements/sprinkles;* ***4****. Other (specify;* ***99****. Don’t know*

***Preliminary analysis:***

Number of correct responses ______

Knows /Doesn't know ___ *(1= Does not know; 2= Knows [at least 1 correct response])*

**Marks** *(0= Does not know; 2= Knows)* **_____**

**K.6** Can you name examples of foods of animal origin that are rich in vitamin A? (***Probe)*** (list all) **_______**

***Animal-source foods***

***61*** *Liver* ***62*** *Kidney* ***63****.Heart* ***64****.Egg yolks/egg from chicken, duck, guinea fowl or other bird* ***65****.Milk, cheese, yogurt or other dairy product*

***Preliminary analysis***

Number of correct responses ______

Knows/Doesn`t know ______ *(1= Does not know; 2= Knows [at least 2 correct responses])*

**Marks** *(0= Does not know; 2= Knows)* **______**

**K.7** Can you name examples of vegetables that are rich in vitamin A? (list all) **_______**

***Green leafy vegetables***

***71****. Amaranths* ***72.*** *Spinach* ***74****. Cassava leaves* ***75****. Pumpkin leaves* ***36****. Kale* ***76****. Other green leafy vegetables (e.g., locally available green leafy vegetables)*

***Preliminary analysis***

Number of correct responses ______

Knows/Doesn`t know ______ *(1= Does not know; 2= Knows (at least 3 correct responses)*

**Marks** *(0= Does not know; 2= Knows)* **______**

**K.8** Can you name examples of fruits that are rich in vitamin A? (list all) _________

***Fruits (orange- or yellow-colored non-citrus fruits)***

***81****.Ripe mango* ***82****.Ripe papaya* ***83****.Pumpkin* ***84****. Cantaloupe/mush melon* ***85****. Water melon* ***86****. Apricot. 87. Other locally available vitamin-A-rich fruits.*

***Preliminary analysis***

Number of correct responses ______

Knows/Doesn`t know ______ *(1= Does not know; 2= Knows [at least 2 correct responses])*

**Marks** *(0= Does not know; 2= Knows)* **______**

**K.9** Can you name examples of roots/tubers that are rich in vitamin A? (list all)____ _________

***Orange-yellow colored roots/tubers***

***91****.Orange sweet potato* ***92****.Carrot* ***93****. Locally available orange-yellow-colored root crops*

***Preliminary analysis***

Number of correct responses ______

Knows/Doesn`t know ______*(1= Does not know; 2= Knows (at least 2 correct responses)*

**Marks** (*0= Does not know; 2= Knows)* _____

**K.10** Can you name examples of foods to which vitamin A has been added from the factory? ______

***Foods fortified with vitamin A***

***10****. Margarine* ***11****.Cooking oil* ***12.*** *Flour* ***13****.Other (specify)* ***99****. Don’t know*

***Preliminary analysis***

Number of correct responses ______

Knows/Doesn`t know ______*(1= Does not know; 2= Knows (at least 1 correct response)*

Marks (0= Does not know; 2= Knows) _____

**Total Knowledge score: ___________**

**___________%**

**SECTION C: Attitude towards Vitamin A-rich foods and Vitamin A deficiency**

**A.1.1** Do you think your child is likely to lack enough of vitamin A in his/her body? ***If likely at all, skip to A.2.1*** ____ **_______**

*-2 = very unlikely -1=unlikely 0=not sure 1= likely 2. Very likely*

***A.1.2*** *If unlikely or very unlikely*: Can you tell me the reason why?

___________________________________________________________________________________________________________________________

**A.2.1**. How serious do you think a lack of vitamin A is/can be to your child? ***If serious at all, skip to A3.1*** ______ **_______**

***-2****=not serious at all* ***-1****. Not serious* ***0****= not sure* ***1****=Serious* ***2****= very serious*

**A.2.2** If Not serious: Can you tell me the reason why it is/may not be serious?

______________________________________________________________________________________________________________

**A.3.1** How important do you think it is to prepare meals with vitamin-A-rich foods such as carrots, green leafy vegetables, sweet potatoes or liver? ***If important at all, skip to A.4.1* _______**  _______***-2****=Not important at all* ***-1****=Not important* ***0****= not sure* ***1****= important 2=very important*

**A.3.2** If Not important: Can you tell me the reasons why it is not important?

______________________________________________________________________________________________________________

**A.4**.1How difficult is it for you to prepare meals with vitamin-A-rich foods? ***If 1 or 2, skip to A5.1* _______**

***-2****=very difficult* ***-1****=difficult* ***0****=so, so 1=Not difficult* ***2****=Not Difficult at all.*

**A.4.2** If Difficult: Can you tell me the reasons why it is difficult?

**_**__________________________________________________________________________________________________________________________*__________________________*

**A.5.1** To what extent do you feel you are able to prepare meals with vitamin A-rich foods? ***If 1 or 2, skip to A.6.1***  ______

*-****2****= Very small extent* ***-1****=Small extent* ***0****= Ok/so-so* ***1****=Large extent* ***2****=very large extent*

**A.5.2** If small or very small extent in 5.1: Can you tell me the reasons why you feel you are not able enough?

________________________________________________________________________________________________________________

**A.6.1** How much do you like the taste of the following foods?

Green leafy vegetables **_______** ____

Orange-fleshed sweet potato **_______** ____

Carrots **_______** ____

Pumpkins **_______** ____

*-2=Dislike strongly -1=dislike 0=Neutral 1. Like slightly 2=like greatly*

**Average score = _______**

**A.7.1** There are things/situations/events/occurrences you can use to know you need to prepare and give more VA rich foods to your child. Do you: ***If you disagree at all, skip to A.8.1*** **_______**

***-2****. Strongly disagree?* ***-1****. Disagree?* ***0****. Neither agree nor disagree?* ***1****. Agree?* ***2****. Agree strongly?*

**A.7.2** If you agree, what do you think can make you know you need to prepare and give more vitamin A-rich foods to your child? *(List all*) **_______**

***3****. Child falls sick;* ***4.*** *Child struggles to see;* ***5****. Child gets eye problems;* ***6****. Child’s gets a skin problem;* ***7****. Child gets diarrhea;* ***8****. Child gets measles;* ***9****. When green vegetables are rare;* ***10****.When they talk about vitamin A;* ***11****.When they talk about child health days;* ***12****. Other (specify)*

**A.8.1** There is nothing that can prevent you from preparing and giving vitamin A-rich foods to your child. ***If 1 or 2, skip to A.8.2*** **_______**_

***-2****. Strongly disagree* ***-1****. Disagree* ***0****. Neither agree nor disagree* ***1****. Agree* ***2****. Agree strongly*

**A.8.2** If you disagree at all, what can prevent you from preparing and giving vitamin A-rich foods to your child? (List all)  **_______**

***3.*** *Lack of knowledge about VA rich foods* ***4****. High cost of VA-rich foods* ***5.*** *Child does not like VA-rich foods* ***6****. Unavailability of VA-rich foods* ***7****. Culture forbids VA-rich foods* ***8****. Lack of skills to prepare VA-rich foods* ***9****. Not applicable (somewhat agrees in 8.1* ***10****. Other (specify)*
